# CRISPR/Cas9 Ablated BCL11A Unveils the Genes with Possible Role of Globin Switching

**DOI:** 10.34172/apb.2023.074

**Published:** 2023-02-21

**Authors:** Fatemeh Movahedi Motlagh, Hamid Reza Soleimanpour‐Lichaei, Mehdi Shamsara, Azadeh Etemadzadeh, Mohammad Hossein Modarressi

**Affiliations:** ^1^Department of Medical Genetics, Tehran University of Medical Sciences, Tehran, Iran.; ^2^Department of Stem Cells and Regenerative Medicine, National Institute of Genetic Engineering and Biotechnology, Tehran, IR Iran.; ^3^Animal Biotechnology Group, Department of Agricultural Biotechnology, National Institute of Genetic Engineering and Biotechnology, Tehran, Iran.

**Keywords:** CRISPR/Cas9, Beta hemoglobinopathies, Fetal hemoglobin, Globin switching, BCL11A knockdown

## Abstract

**Purpose:**

Fetal hemoglobin (HbF) upregulation is a mitigating factor in β-hemoglobinopathies therapy like β-thalassemia and sickle cell diseases. Finding molecular mechanisms and the key regulators responsible for globin switching could be helpful to develop effective ways to HbF upregulation. In our prior *in silico* report, we identified a few factors that are likely to be responsible for globin switching. The goal of this study is to experimentally validate the factors.

**Methods:**

We established K562 cell line with BCL11A knock down leading to increase in HBG1/2 using CRISPR/Cas9 system. Then, using quantitative polymerase chain reaction (qPCR), we determined the expression level of the factors which were previously identified in our prior *in silico* study.

**Results:**

our analysis showed that BCL11A was substantially knocked down, resulting in the upregulation of HBG1/2 in the BCL11A-ablated K562 cells using CRISPR/Cas9 system. Additionally, the experimental data acquired in this study validated our prior bioinformatics findings about three potentially responsible genes for globin switching, namely HIST1H2Bl, TRIM58, and Al133243.2.

**Conclusion:**

BCL11A is a promising candidate for the treatment of β-hemoglobinopathies, with high HbF reactivation. In addition, HIST1H2BL, TRIM58 and Al133243.2 are likely to be involved in the mechanism of hemoglobin switching. To further validate the selected genes, more experimental *in vivo* and *in vitro* studies are required.

## Introduction

 One of the most common inherited hemoglobin disorders is thalassemia.^1^ Beta thalassemia patients depend on long term treatment, however they are still associated with morbidity and mortality.^2^ Bone marrow (BM) transplantation is by far the most definitive treatment for these patients. However, there are many problems associated with transplantation, including limited appropriate donors with identical HLA. Donors may encounter complications such as transplant rejection, and immunoglobulin reaction of host cells (graft-versus-host disease, GVHD).^3^ Therefore, gene therapy utilizing patients’ own hematopoietic stem cells is a preferable approach.^4^ Autologous gene edited hematopoietic stem cells (HSCs) is a definitive cure that can overcome the problems of finding BM compatible donors and GVHD.^5^ One of the therapeutic approaches for the edition of HSCs is to increase fetal hemoglobin (HbF) expression which can lead to the amelioration of hemoglobinopathies symptoms.^6^

 In β-thalassemia, HbF reactivation could compensate for the β-globin deficiency and prevent the accumulation of extra unmatched α-globin chains. Various gene editing strategies have been applied to induce HbF expression as a treatment for hemoglobinopathies. BCL11A was recognized as the master repressor of HbF production by genome-wide association studies, and several studies have validated its down-regulation effect on increased HbF levels.^7,8^ However, genetic editing of BCL11A coding site could not be an appropriate treatment method, because its coding site has a crucial role in the hematopoietic stem cell function and the formation of lymphoid lineage. Fortunately, recent research found that disrupting the GATAA motif in the BCL11A erythroid enhancer resulted in a significant increase in HbF expression.^9^ Fortunately, recent studies revealed that the disruption of the GATAA motif in BCL11A erythroid enhancer led to a substantial increase in HbF expression.^10^

 Since re-activation of HbF expression in adult erythroid cells might be a promising tool for the hemoglobin disorders therapy, it is clinically significant to discover the transcriptional regulation of globin switching. The purpose of this research is to survey the genes identified through our earlier *in silico* studies and introduced in our most recent publication.

 For this aim, we initially established a K562 cell line with knocked-down BCL11A by “clustered regularly interspaced short palindromic repeats (CRISPR) RNA-guided nucleases” (CRISPR/Cas9 system). Then we evaluated the expression of putative key genes found by *in silico* studies, including HIST1H2BL, AL133243.2, TRIM58, FAM210B, RPS27A, and BPGM.

## Materials and Methods

###  sgRNA designing and cloning

**Figure 1:**
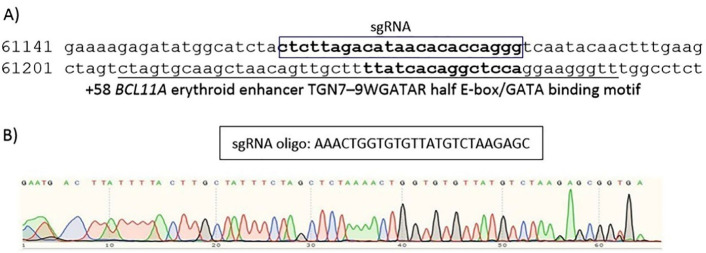
Information of our sgRNA. (A) The position of the sgRNA used in the study. (B) Confirmation of the presence of inserts using sanger sequencing

 Using CRISPOR, we designed a gRNA. Considering the efficiency and off-target scores, a gRNA was selected to target erythroid enhancer of BCL11A. The position of the sgRNA used in this study are shown in Figure 1. The oligonucleotides of sgRNA, top and bottom strand, were made double stranded and then ligated into a PX458 plasmid (pSpCas9(BB)-2A-GFP, Addgene, Cat no: 48138) co-expressing *Streptococcus* pyogenes Cas9 protein (SpCas9) and green fluorescent protein. The confirmation for the presence of the inserts was performed by polymerase chain reaction (PCR) and the sequencing of the PCR products by Sanger’s method. The vectors were amplified in DH5α competent cells and the purification of the plasmids was performed using the QIAGEN Plasmid Plus Midi Kit.

###  Cell culture

 The cell line K562 was supplied from the Iranian Biological Resource Center, Tehran, Iran, and then cultured under RPMI 1640 medium containing 1% penicillin/streptomycin (Sigma-Aldrich, USA), 10% FBS, and at 37 °C/5% CO2.

###  DNA transfection by electroporation and flowcytometry

 Using Bio-Rad, we electroporated 5 × 10^6^ K562 cells with 12 µg of recombinant pX458. Electroporation settings for this cell line were 2649, 1050 μF, and 1 pulse. DNA delivery into cells was examined using Flow cytometry 48 hours after transfection. Flow cytometry analysis (FACSCalibur, BD Biosciences) was conducted based upon the instructions from the manufacturer and analysis of all data were performed by Cell Quest or FlowJo (BD Biosciences).

###  RNA extraction and quantitative RT-PCR

 The extraction of total RNA was performed by RNX-Plus kit (SINAGENE, Iran) based upon the protocol from the manufacturer. 2–5 µg RNA was reverse transcribed using the BIONEER (k-2101) Reverse Transcription kit. Quantitative PCR analysis was performed in triplicate using primers listed in Table 1 using RealQ Plus 2x Master Mix Green (Amplicon) and analyzed by a LightCycler^®^ 96 System (Roche Molecular Systems). The target genes’ threshold cycles (Ct) were obtained from LightCycler^®^ 96 System Software and were normalized by B2M.

**Table 1:** Primers used for quantitative real-time PCR

**Gene**	**Sequence of primer (5’- 3’)**	**PCR Product length (bp)**
BCL11A(FP)	AACCCCAGCACTTAAGCAAA	114
BCL11A(RP)	GGAGGTCATGATCCCCTTCT	
HBG(FP)	TGGATGATCTCAAGGGCAC	209
HBG(RP)	TCAGTGGTATCTGGAGGACA	
HIST1H2BL-FP	AAGGCCGTCACCAAGTACAC	124
HIST1H2BL-RP	CCCCAGTGATAGGAAGAGCG	
AL133243.2-FP	ACACAGTTGTGCATACAGCTA	191
AL133243.2-RP	TCCACTGTAATTCCTTTGGCTCA	
TRIM58-FP	AGAGGAGTCCTGAGCAGAAGTA	143
TRIM58-RP	GTGGCGGGATCCAGCTTTAC	
FAM210B-FP	AGCGTCAGCTGCACAGAG	158
FAM210B-RP	GGCATGTCCACACCACTTGA	
RPS27A-FP	CAAGATCCAGGATAAGGAAGGAAT	147
RPS27A-RP	GCACCACCACGAAGTCTCAA	
BPGM-FP	AAAACACCTGGAAGGTATCTCA	96
BPGM-RP	GCACGCAGGTTTTCATCCAA	

###  Statistical analysis

 The analysis of relative gene expression was done by the formula 2^−ΔΔCT^ method using Ct value. The error bars represent the standard deviations. Data were analyzed using two group *t* test. *P* < 0.05 was used to determine statistical significance (**P value < 0.01 ***P value < 0.001 ****P value < 0.0001). GraphPad Prism 5 was used for calculations (GraphPad Software, San Diego).

## Results and Discussion

 Detecting key genes involved in the globin switching and knowing their interactions helped us find more evidence for its molecular mechanisms.

**Figure 2:**
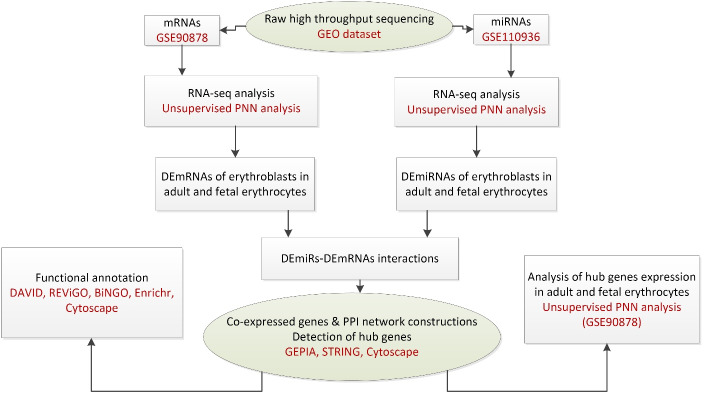
The flow diagram of bioinformatics study.^11^

**Figure 3:**
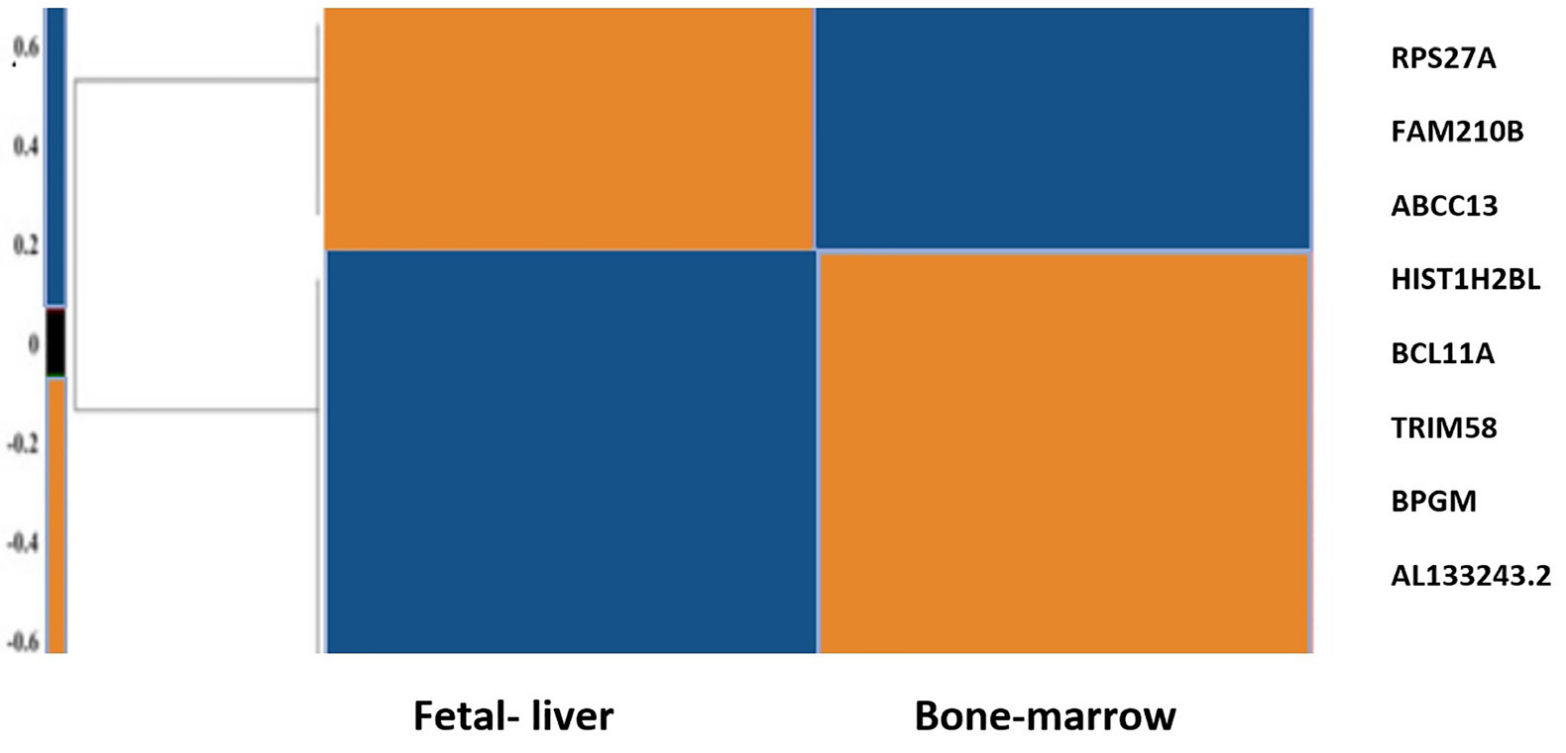
Heat map of the hub genes. mRNA expression was normalized in the range of -3 to 3. The down-regulated and up-regulated genes are colored blue and orange, respectively.^11^

 In our prior study, we used bioinformatics and systems biology methods to introduce probable hub genes that play roles in the globin switching process. For this aim, RNA-seq data analysis was used to define DEmRNAs (differentially expressed mRNAs) and DEmiRs (differentially expressed miRNAs) in adult and fetal erythroblasts. Moreover, we used multiple databases to construct co-expression and protein protein interaction (PPI) networks to find key mRNAs, as depicted in the flow diagram of our study in Figure 2. The hub genes included Histh2bl, Al13243.2, Trim58, Bpgm, and Fam210b in the coexpression network, as well as RPS27A in the PPI network. In addition, we surveyed the expression changes of the hub genes in adult and fetal erythrocytes using RNA-seq analysis of the NCBI Gene Expression Omnibus (GEO) samples. As indicated in Figure 3, the expression of the bcl11a, rps27a, and fam210b were decreased, while that of the hist1h2bl, al1332433.2, and trim58 were increased in adult erythrocytes. It is obvious that experimental studies are required to consider these genes as key factors in globin switching. In this study, we designed the experiment to confirm the results of our previous *in silico* study. Therefore, we established a K562 cell line with a decrease in BCL11A by CRISPR/Cas9 system. Then, we evaluated the expression of the hub genes found by *in silico* study including HIST1H2BL, AL133243.2, TRIM58, FAM210B, RPS27A, and BPGM.^11^

###  Cloning of gRNAs into PX458

 After annealing sgRNA oligos, they were ligated into PX458 plasmids. Ligated plasmids were transformed into chemically competent bacterial cells and plated on agar plates containing the ampicillin. Clones were picked after 24 hours, inoculated into 5 mL LB broth medium supplemented with ampicillin, and finally cultured overnight. The plasmids were then extracted and the confirmation of the inserts was performed by PCR and subsequent sequencing (Figure 3).

###  Transfection of K562 cell line by electroporation

**Figure 4:**
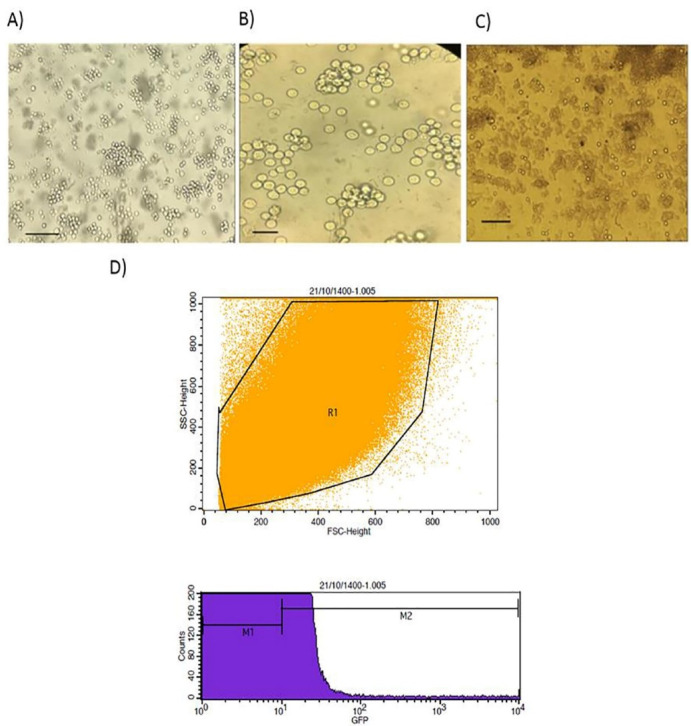
the results of transfection. (A) K562 cell line Bars: 100 µm, (B) K562 cell line Bars: 40 µm, (C) K562 cell line post transfection (G1 cells) Bars: 100 µm, and (D) The results of flow cytometry analysis 48 hours post transfection

 K562 cell line was transfected with PX458 plasmid harboring gRNA using electroporation. Transfection efficiency was determined by flowcytometry analysis after 48 hours. The results of flowcytometry analysis showed that about 12% of the electroporated cells were successfully transfected (Figure 4).

###  Disruption of the BCL11A enhancer upregulated HBG1/2 expression in K562 cell lines

**Figure 5:**
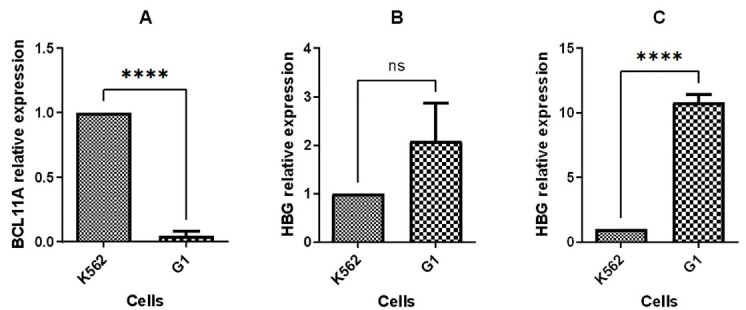
Quantitative RT-PCR to compare gene expression in G1 with K562 cell line, (A) BCL11A expression 48 hours post transfection, (B) HBG1/2 expression 48 hours post transfection, and (C) HBG1/2 expression 96 hours post transfection. (**P value < 0.01 ***P value < 0.001 ****P value < 0.0001, ns: not significant)

 48 hours post transfection, expression changes of BCL11A, HBG1/2, and the putative hub genes, which were earlier introduced by our prior *in silico* study, were analyzed. The results showed that the BCL11A expression was substantially decreased (Figure 5).

 Moreover, upregulation of HBG1/2 was observed with 2-fold and 10-fold increase in mRNA level in BCL11A knocked-down cells compared to non-transfected cells at 48 hours and 96 hours post transfection, respectively (Figure 5).

 Previous studies have validated BCL11A as a key HbF silencing factor that regulates the developmental globin switching.^12^ BCL11A binds to the location at the HBG1/2 gene’s proximal promoters to silence γ-globin gene. Some studies provide detailed information on the mechanism by which BCL11A and its co-factors act.^13,14^ In a recent study, the efficiency of three methods including BCL11A and KLF1 knock down as well as HBG1/2 promoter editions was compared. The findings suggest that knocking down KLF1 may interfere with the expression of cell-cell interaction genes like ITGA2B and CD44, microcytosis genes like AQP1, and cancer genes like FLI-1. Furthermore, it was demonstrated that BCL11A knock down was safer than the other methods, with the ability to upregulate HbF more than four folds in BCL11A-edited samples compared to control sample.^15^

 This research, in line with the previous studies, demonstrated that inactivation of BCL11A enhancer, located in the second intron, can stimulate fetal hemoglobin synthesis and could be applied as a possible gene therapy strategy for β-hemoglobinopathies.^16^ The erythroid-specific BCL11A enhancer has been suggested as the most demanded candidate for clinical use.^17^ Previous research has suggested that editing based on NHEJ approaches might be more efficient than HDR-based editing.^18^

###  Evaluation of hub genes expression in transfected K562 cell lines

**Figure 6:**
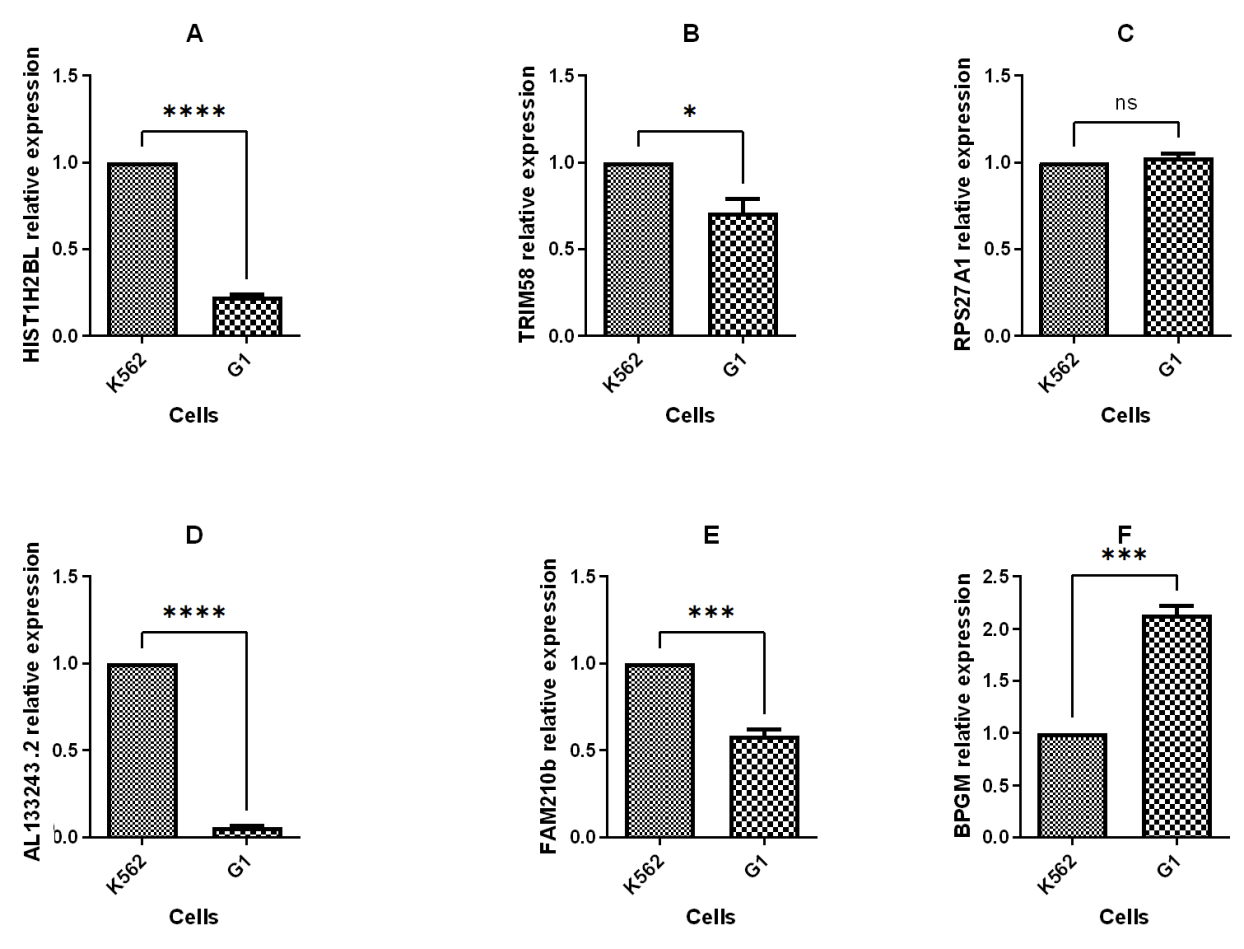
Quantitative RT-PCR to compare hub genes expression in G1 with K562 cell line. (**P value < 0.01 ***P value < 0.001 ****P value < 0.0001, ns: not significant)

 The expression of hub genes found by *in silico* study including HIST1H2BL, AL133243.2, TRIM58, FAM210B, RPS27A, and BPGM was evaluated using q-PCR 48 hours post transfection. The q-PCR analysis revealed that BPGM was upregulated, while HIST1H2BL, AL133243.2, TRIM58, and FAM210B were down regulated, and RPS27A expression remained unchanged after transfection (Figure 6). The q-PCR results and bioinformatics data for three genes, including HIST1H2BL, TRIM58, and AL13243.2, were consistent.

 TRIM58, an E3 ubiquitin protein ligase, has been demonstrated to be crucial in late erythropoiesis.^19^ Previous research identified HIST1H2BL, which encodes a member of the H2B histone family, as a target gene for the BCL11A transcription factor. Furthermore, it was demonstrated that HIST1H2BL is a regulator of transcription using chromatin organization. This information implies that other transcriptional regulatory mechanisms, such as chromatin architecture, may potentially control the production of HbF.^11,20^

 AL133243.2 is an lncRNA. It has been established that gene expression is regulated by lncRNAs via DNA, RNA, and protein interactions.^21^ LncRNAs have many important functions in erythropoiesis, like globin switching.^22^ In a previous study we demonstrated that AL133243.2 may act as a regulator at the transcriptional and translational levels.^11^

## Conclusion

 To obtain insights into the globin switching process, we have used bioinformatics and experimental approaches to find the probable key genes. This approach enabled us to identify some genes that may increase HbF levels.

 In summary, we have demonstrated that BCL11A knock down can be used to induce HBG1/2 expression to therapeutically relevant levels. In comparison to globin gene addition, genome editing approach to obtain globin reverse switch would have the benefit of high-level production of endogenous γ-globin.^18^

 In a prior bioinformatics study, we identified six genes with potential key roles in globin switching; however, in the current study only three genes (out of the six genes) were experimentally validated for their possible function in globin switching.^11^

 The safety and specificity of gene editing are critical parameters for clinical use of therapeutic tools.^17^ Our results reveal the function of an lncRNA, AL133243.2, in the HbF production. Defining the role of lncRNA in the post-transcriptional control of HbF may prompt the investigation of therapeutic elements that would prevent widespread alterations to the transcriptome.

 Overall, our findings have identified potential targets for therapeutic reactivation of fetal hemoglobin. The clinical application of the genes involved in HBG reactivation requires further *in vitro* and *in vivo* research.

## Acknowledgments

 This work was supported by Tehran University of Medical Sciences, Tehran, Iran.

## Competing Interests

 The authors have no conflict of interest.

## Ethical Approval

 This study approved by the ethics committiee of Tehran University of Medical Sciences approved the study (Code: IR.TUMS.MEDICINE.REC.1398.762).
